# The effect of light therapy on sleep disorders and psychobehavioral symptoms in patients with Alzheimer’s disease: A meta-analysis

**DOI:** 10.1371/journal.pone.0293977

**Published:** 2023-12-06

**Authors:** Lili Zang, Xiaotong Liu, Yu Li, Jiang Liu, Qiuying Lu, Yue Zhang, Qinghui Meng

**Affiliations:** School of Nursing, Weifang Medical University, Weifang, Shandong Province, China; Technical University of Munich, Germany, UNITED KINGDOM

## Abstract

**Background:**

Although Alzheimer’s disease (AD) mainly affects cognitive function, it is often accompanied by sleep disorders and psychobehavioral symptoms. These symptoms, including depression, agitation, and psychotic symptoms, are prominent hospitalization causes among patients with AD. Currently, relatively more research exists on light therapy for sleep disorders, while those on psychobehavioral symptoms are gradually increasing. However, no consensus exists on these results because of the vulnerability of light therapy to multiple factors, including light intensity and duration. Thus, further research investigating this aspect is warranted.

**Objective:**

To evaluate the efficacy of light therapy in improving sleep disorders and psychobehavioural symptoms in patients with AD.

**Methods:**

In this meta-analysis, relevant literature was searched in Embase, the Clinical Trials Registry, Web of Science, PubMed, and the Cochrane Library up to December 2022. Furthermore, a fixed-effects model was used for data analysis.

**Results:**

Fifteen randomized controlled trials involving 598 patients with AD were included. In the case of sleep disorders, our meta-analysis revealed that light therapy significantly improved sleep efficiency (MD = −2.42, 95% CI = −3.37 to −1.48, *p* < 0.00001), increased interdaily stability (MD = −0.04, 95% CI = −0.05 to −0.03, *p* < 0.00001), and reduced intradaily variability (MD = −0.07, 95% CI = −0.10 to −0.05, *p* < 0.00001). With respect to psychotic behavior, light therapy was found to alleviate depression (MD = −2.55, 95% CI = −2.98 to −2.12, *p* < 0.00001) as well as reduce agitation (MD = −3.97, 95% CI = −5.09 to −2.84, *p* < 0.00001) and caregiver burden (MD = −3.57, 95% CI = −5.28 to −1.87, *p* < 0.00001).

**Conclusion:**

Light therapy leads to significant improvement in sleep and psychobehavioral symptoms and is associated with relatively fewer side effects in patients with AD, indicating its potential as a promising treatment option for AD.

## Introduction

Alzheimer’s disease (AD) is a neurodegenerative disorder exhibiting gradual disease progression [1]. Cognitive decline represents the most prominent clinical manifestation of AD, it is often accompanied by sleep disturbances and varied psychobehavioral symptoms, including apathetic and depressive behavior as well as agitated behavior characterized by exaggerated motor activity and verbal and/or physical aggression [2,3]. Approximately 90% of patients develop one or more psychobehavioral symptoms during the AD course [4,5]. Moreover, patients in advanced AD stages may exhibit severe cognitive impairment, behavioral disturbances, and high caregiver dependence, possibly reducing the quality of life of these patients, worsening the difficulty of caregiving, and increasing the healthcare burden [6].

Sleep disorders are common in AD, with 70% of patients experiencing sleep disruption in the early stages [7]. The suprachiasmatic nucleus (SCN), a sleep regulator in the brain’s hypothalamus [8], synchronizes the circadian rhythm by regulating the light-dark and sleep-wake cycles [9]. In patients with AD, reduced light input due to reduced outdoor exposure and poor sensitivity to light stimuli owing to neuropathic damage can lead to decreased circadian rhythm stability [10]. Additionally, reduced social interaction and age-related eye defects (e.g., macular degeneration and cataracts) may also affect light transmission in the eyes, leading to circadian rhythm and sleep disorders [11].

The primary treatment for AD involves pharmacological therapy, including cholinesterase inhibitors, NMDA receptor antagonists, Aβ inhibitors, and intestinal flora modulators [12]. Although medication may alleviate AD-induced cognitive and memory impairments to some extent, it cannot stop the progression or cure this neurodegenerative disease. Moreover, some patients with AD may develop medication-related side effects, such as poor appetite, diarrhea, and hallucinations [13–15]. Therefore, a highly efficacious treatment with no side effects is currently a hot spot and challenging research area.

Photobiomodulation (PBM) is a non-pharmacological therapy employing light energy to modulate biological function and promote therapeutic effects. PBM stimulates the SCN with varying light intensities and durations to regulate melatonin secretion and facilitate the communication between the hypothalamus and cortex [16], ultimately modulating circadian rhythms. Light therapy can also shorten sleep latency, reduce nocturnal insomnia, increase total sleep time, and improve sleep quality in patients with AD [17,18]. Recently, research on applying PBM in neurology has risen rapidly, particularly concerning the treatment and regulation of neurodegenerative diseases [19]. Studies have shown that PBM can improve cognitive function, enhance the quality of life, and reduce caregiver burden in patients with AD by reducing neuronal damage and inflammatory response [20].

Despite light therapy receiving increasing attention as a potential non-pharmacological intervention for AD, a systematic evaluation of the efficacy and safety of this treatment for patients with AD is still unavailable. To fill this knowledge gap, a comprehensive systematic review and meta-analysis is required to more rigorously evaluate the effectiveness of light therapy in ameliorating sleep disorders and psychobehavioral symptoms among patients with AD.

A meta-analysis by Van Maanen et al. [21] used broad study inclusion criteria and included those involving patients with reported or diagnosed sleep disorders, showing that light therapy was effective in treating sleep problems. Similarly, Roccaro et al. [22] conducted a systematic review of research examining the effect of light therapy in alleviating sleep and rhythm disorders in patients with AD, concluding that light therapy ameliorates circadian rhythm disturbances and sleep efficiency (SE) in patients with AD. Furthermore, previous studies have indicated that light therapy can reduce cognitive decompensation and depression behavior in patients with AD [23,24], however, further research is warranted to add to the scarce evidence on its usefulness. Compared with prior meta-analyses, our meta-analysis had a relatively larger number of included articles and outcome metrics, expanded the sample size, and only included randomized controlled trials (RCTs) to reduce sampling error. Therefore, our meta-analysis may strengthen the evidence on the effectiveness of light therapy in treating sleep disorders and psychobehavioral symptoms in patients with AD.

## Materials and methods

The systematic review protocol used in this research was according to the Preferred Reporting for Items for Systematic Reviews and Meta-Analyses 2020 (PRISMA) criteria to guide the reporting of results [25]. The PRISMA checklist is shown in S1 Table. The protocol for this systematic review was registered in the PROSPERO database (registration number: CRD42023406390).

### Literature search strategy

To conduct this meta-analysis, we performed electronic searches in various databases until December 2022 under the guidance of a library search specialist to identify all RCTs that were related to light therapy intervention for AD or dementia. The subject terms used in the search included “Alzheimer Disease,” “Phototherapy,” “Sleep Disorders,” and “Cognition Disorders,” while free terms comprised “Dementia,” “AD,” “Light Therapy,” “Sleep Wake Disorder,” “Disorder,” and “Cognition.” For a complete list of the used keywords, see S2 Table. The searched databases encompassed Embase, the Clinical Trials Registry, Web of Science, PubMed, and the Cochrane Library. We searched for the keywords described above in each database with separate title, keywords, and abstract filters for each search string. The reference lists of all relevant studies in previous reviews were examined to avoid potential omissions.

### Inclusion and exclusion criteria

Inclusion criteria for selected studies:

1. Patients were older adults (aged 60–85 years) diagnosed with AD by health care institutions and had Mini-Mental State Examination (MMSE) scores between 6 and 26.
2. Study design was RCT with complete data and results.
3. Patients in the intervention group had undergone light therapy, whereas those in the control group had received dim light or usual care.
4. Patients who were not sensitive to light.
5. Studies had at least one of the following outcome indicators: SE, interdaily stability (IS), intradaily variability (IV), Pittsburgh sleep quality index (PSQI), relative amplitude (RA), or wake after sleep onset (WASO) or at least one of the following instruments: the Alzheimer’s Disease Assessment Scale-Cognitive subscale (ADAS-cog), Cohen-Mansfield agitation inventory (CMAI), Cornell Scale for Depression in Dementia (CSDD), Zarit Caregiver Burden Interview (ZBI), Neuropsychiatric Inventory (NPI), or MMSE.

Exclusion criteria for selected studies:

1. Studies including patients who regularly use valium, antidepressants, or sleeping pills.
2. Studies involving patients with eye diseases (e.g., glaucoma, blindness, or cataracts) or other neurological disorders (e.g., Parkinson’s disease or intellectual disability).

### Study selection

All references were stored and managed in EndNote X9. The included studies were screened by two independent reviewers according to the title and abstract. The extracted results were then cross-checked for accuracy and consistency, and all eligible studies were retained for full-text evaluation. Next, two researchers independently extracted and stored the data in the Review Manager software. In the case of controversial literature, the study inclusion was decided by the research team after discussion. To ensure the completeness of our systematic review, only articles for which the full text was available were included. Furthermore, we collected the following data (if available) from each study: first author’s name, publication year, publication country, sample size, gender, the type of light therapy, pre-intervention MMSE score, and the data of the outcome indicators. To avoid potential errors, two researchers assessed the data before analysis. Finally, all relevant information was extracted to the standard template by two reviewers independently.

### Assessment of quality and risk of bias

Two researchers used the risk bias assessment tool in the Cochrane Handbook of Systematic Reviews of Interventions (version 5.1.0) [26] to evaluate the included literature in terms of high, unclear, and low risk of bias based on random sequence generation, allocation concealment, blinding of participants and performers, outcome evaluator, study data integrity, selective outcome reporting, and other sources of bias. Each study was evaluated using “yes (low bias),” “unclear (lack of relevant information or uncertainty about bias),” or “no (high bias)” responses for the seven items mentioned above. The risk of bias was determined by two researchers independently. Moreover, if any disagreement arose, a third researcher was asked to help resolve the debate.

### Outcome measures

A total of 12 outcome indicators were included in this meta-analysis: SE, IS, IV, PSQI, RA, WASO, ADAS-cog, CMAI, CSDD, ZBI, NPI, and MMSE.

### Statistical analysis

We used Review Manager (RevMan) version 5.3 software (The Nordic Cochrane Center, The Cochrane Collaboration, Copenhagen, Denmark) to combine the results of the various studies and assess the overall effect of light therapy. Mean difference (MD) was used as a summary statistic in the trials that assessed the same outcome and measure using a consistent method [27]. Furthermore, MD and 95% confidence intervals (CIs) were employed to represent the outcomes because all outcomes were continuous, wherein MD was calculated as treatment-control. The heterogeneity was evaluated by *I*^*2*^ statistics and expressed as the percentage of variation across studies. In this test, an *I*^*2*^ value of <50% indicates no heterogeneity, while an *I*^*2*^ value of >50% demonstrates a high degree of heterogeneity. *P* < 0.05 was considered statistically significant. Furthermore, we performed subgroup analysis to explore the sources of heterogeneity across the included studies. Finally, funnel plots were used to evaluate the included literature for publication bias.

## Results

**Fig 1:**
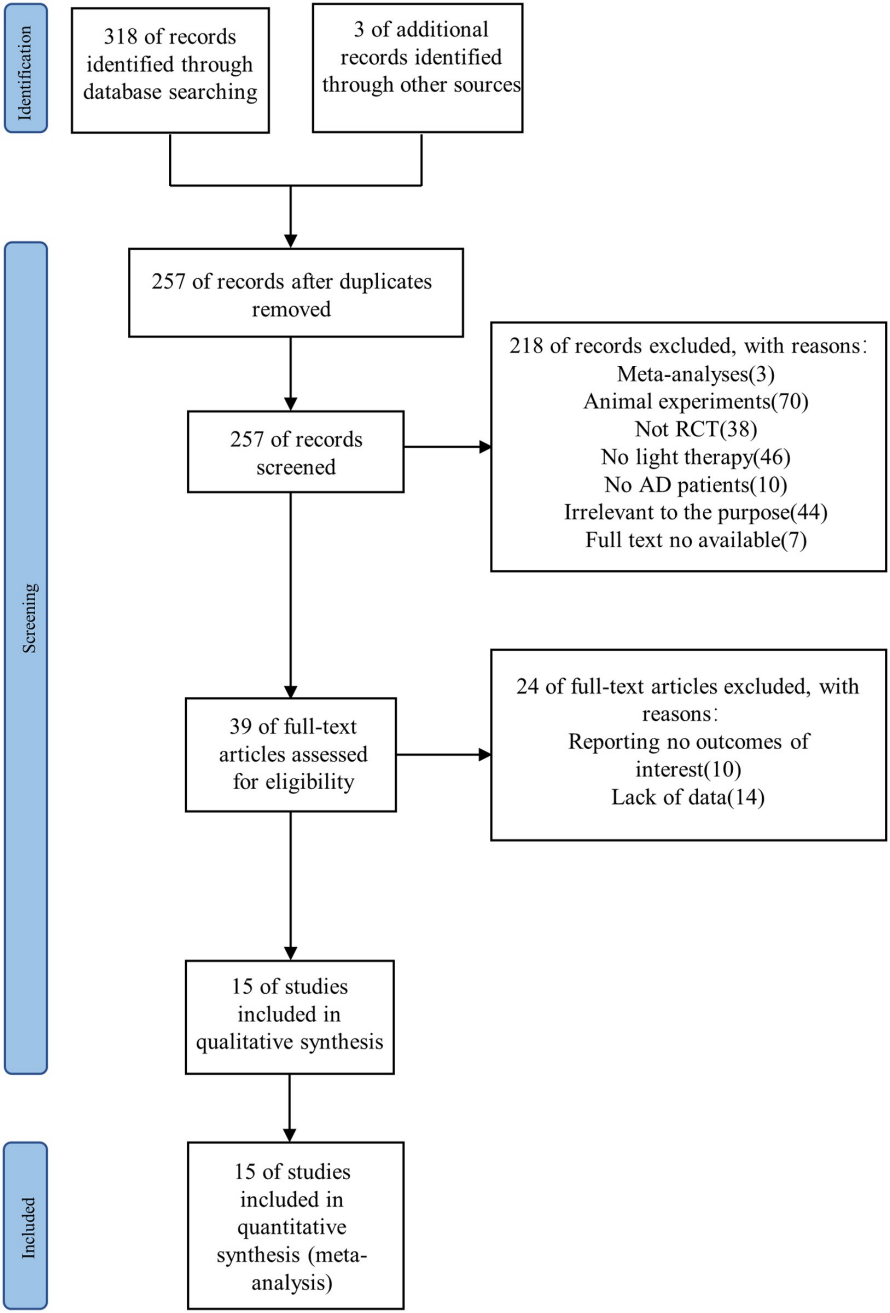
Flow chart of the study selection process.

The study selection process is illustrated in Fig 1. A total of 318 articles were filtered via the database search, along with three relevant studies that were included after applying the snowballing method. Further, 257 articles remained after the removal of duplicate articles. According to the screening process of the title and abstract of the articles, 218 articles were excluded due to the following reasons: 38 articles were not RCTs, three were meta-analyses, 70 were animal experiments, 46 did not use eligible interventions, 10 did not involve eligible populations, seven did not have full-text availability, and 44 had irrelevant research objectives. Furthermore, we screened the full text of the remaining 39 articles, after which 10 were excluded because they did not have the required outcome indicators and 14 were removed due to incomplete data. Finally, a meta-analysis of 15 studies (encompassing 598 patients with AD) was conducted [28–42] to investigate the effects of light therapy versus usual care on sleep disorders and psychobehavioral symptoms in patients with AD.

### Characteristics of included studies

All 15 included studies were written in English and published between 2005 and 2022. The characteristics of these studies are summarized in Table 1, while the summary of the applied interventions is provided in Table 2. The included studies were performed in seven different countries, where eight were conducted in the United States [28,31–33,36,37,40,41], two in the UK [29,38], and one each in Korea [35], France [34], China [30], the Netherlands [39], and Switzerland [42]. Additionally, nine studies [28,29,33,35–37,39,41,42] included patients with MMSE scores indicating mild to moderate AD, whereas six [29–32,38,40] involved those having MMSE scores suggestive of moderate to severe AD. All studies were RCTs, and the interventions investigated dealt with applying light therapy devices or light therapy. Four studies examined the application of light therapy devices [28,34,36,37], while eleven involved light therapy [29–33,35,38–42]. Lastly, two studies were cross-designed [33,40].

**Table 1:** Characteristics of included RCT studies.

First author	Country	Year	Design	Setting	Light therapy (I)	Control (C)	Number of patients (I/C)	Gender (Male/Female)	Age in years (mean ± SD) I/C	MMSE score	Outcomes
Guillaume	France	2022	2-arm RCT	Nursing home	PBM medical device	All NIR emissions were inactivated	53 (27/26)	I: 12/15 C: 10/16	72.4 ± 7.0/73.7 ± 6.4	20.5 ± 3.6	MMSE, ADAS-cog
Dowling (a)	USA	2005	3-arm RCT	LTC facilities	Bright light	Usual indoor light	46 (29/17)	36/10	84 ± 10	7 ± 7	SE, RA, IS, IV
Dowling (b)	USA	2005	3-arm RCT	LTC facilities	I_1_: Morning bright light I_2_: Afternoon bright light	Usual indoor light	70 (I_1_: 29, I_2_: 24/17)	13/57	84 ± 10	7 ±7	SE
Linda L. Chao	USA	2019	2-arm RCT	LTC facilities	Home PBM treatment	Usual care	8 (4/4)	I: 1/3 C: 2/2	80.5 ± 6.5/79.0 ± 5.9	19.5 ± 7	ADAS-cog, NPI
Friedman	USA	2012	2-arm RCT	Community	Morning bright light	Dim red light	54 (31/23)	I: 20/11 C: 13/10	77.9 ± 8.1	22.1 ± 4.7	WASO, SE
Linda Chao	USA	2020	2-arm RCT	Community	Immediate PBM treatment	Delayed PBM treatment	10 (6/4)	I: 3/3 C: 2/2	78.3 ± 10.2/75.0 ± 10.8	19.5 ± 7	ADAS-cog, NPI
Linda Chao	USA	2022	2-arm RCT	Community	Transcranial and intranasal NIR light	No stimulation	14 (7/7)	I: 3/4 C: 3/4	68.1/72.4	23.4 ± 7.1	ADAS-cog, NPI
Chenjun Zou	China	2022	2-arm RCT	Nursing home	Light therapy	Dim light	61 (34/27)	I: 16/18 C: 12/15	75.94 ± 9.47/73.04 ± 9.34	9.18 ± 4.56	NPI, ZBI
Vivien Bromundt	Switzerland	2019	2-arm RCT	Nursing home	Morning bright light	Dim light	20 (10/10)	3/17	85.6 ± 5.8	13.15 ± 10.3	IV, IS, RA, SE, CMAI
Burns	UK	2009	2-arm RCT	Nursing home	Bright light therapy	Standard light	48 (22/26)	I: 6/16 C: 10/16	84.5 ± 8/82.5 ± 7.6	6.9 ± 5.3	CMAI, MMSE
Figueiro	USA	2019	2-arm crossover RCT	LTC facilities	Whole-day bright light	Dim light	46 (43/44)	32/14	85.1 ± 7.1	14.7 ± 4.3	PSQI, CSDD, CMAI, IS, IV, SE
Kim	Korea	2021	2-arm RCT	Community	Blue-enriched white light	Blue-enriched white light + blue-attenuating sunglasses	25 (14/11)	I: 2/12 C: 5/6	77.36 ± 5.79/78.55 ± 7.71	16.6	CSDD, ZBI, SE, WASO, PSQI, MMSE
Riemersma-van der Lek	Netherlands	2008	4-arm RCT	Nursing home	I_1_: Bright light I_2_: Bright light and melatonin I_3_: Melatonin	Dim light + placebo drug	189 (I_1_: 49, I_2_: 49, I_3_: 46/45)	I_1_: 4/45 I_2_: 2/47 I_3_: 8/38 C: 5/40	I_1_: 85 ± 6 I_2_: 87 ± 6 I_3_: 86 ± 5 C: 85 ± 5	14.4 ± 6.6	CSDD, CMAI, SE, MMSE
Livingston	UK	2019	2-arm RCT	Community	Morning bright light + sleep education	Treatment as usual (no light therapy)	62 (42/20)	I: 9/33 C: 10/10	80.4 ± 9/79.6 ± 7	Very mild, mild, moderate, and severe AD with sleep disorders	NPI, PSQI, ZBI, IS, IV, RA, SE
Sloane	USA	2015	2-arm crossover RCT	Community	Whole-day blue-white light	Red-yellow light	17 (15/16)	6/11	11 out of 17 participants >80	12.7 ± 9.1	IS, IV, SE, ZBI, CSDD

RCT = randomized controlled trial, LTC = long-term care, PBM = photobiomodulation, NIR = near-infrared, SD = standard deviation.

**Table 2:** Summary of light therapy interventions.

First author (Year)	Type of light therapy	Light source/wavelength	Light therapy device	Distance of light from patient	Light intensity	Intervention time	Duration of light therapy	Frequency of light therapy	Total time exposed to light therapy
Guillaume (2022)	Laser diode, LED	Laser diode: 850 nm IR LED: 850 nm Red LED: 660 nm	PBM medical device RGn530	NR	NR	NR	25 min	5 days/week for 8 weeks	Approximately 17 h
Dowling (2005a) [31]	Full-spectrum white light	NR	Light box	1.2 m in front of patient	≥2500 lux	09:30–10:30	1 h	5 days/week for 10 weeks	50 h
Dowling (2005b) [32]	Full-spectrum white light	NR	Light box	1.2 m in front of patient	≥2500 lux	15:30–16:30	1 h	5 days/week for 10 weeks	50 h
Linda L. Chao (2019)	NIR	LED, 810 nm	Transcranial and intranasal LEDs of a PBM device	NR	NR	NR	20 min	3 days/week for 12 weeks	12 h
Friedman (2012) [41]	Full-spectrum white light	NR	Light box	NR	4200 lux	Initiated within 30 min of patient’s wake time	0.5 h	2 weeks	7 h
Linda Chao (2020)	NIR	NR	Transcranial and intranasal NIR light of a PBM device	NR	NR	NR	20 min	4 days/week for 12 weeks	16 h
Linda Chao (2022)	NIR	NR	Transcranial and intranasal NIR light of a PBM device	NR	NR	NR	20 min	4 days/week for 16 weeks	Approximately 21 h
Chenjun Zou (2022)	Full-spectrum white light	NR	Light box	Within 50 cm	1400 lux	09:00–09:30	0.5 h	4 weeks	14 h
Vivien Bromundt (2019)	Full-spectrum white light	LED, NR	Light tubes	NR	0.35–130 lux	90 min of morning light and 90 min of afternoon light	3 h	17 weeks	357 h
Burns (2009) [29]	Full-spectrum BLT	LED, NR	Light box	NR	1000 lux	10:00–12:00	2 h	2 weeks	28 h
Figueiro (2019) [33]	Full-spectrum white light	LED, 555 nm	Light box, light tables, and floor lights	NR	567 lux^a^	From habitual wake-time (06:00–08:00) to 18:00	10–12 h	4 weeks	280–336 h
Kim (2021) [35]	Blue-enriched white light	LED, 400–700 nm	Light box	61 cm in front of patient	30 lux	09:00–10:00	1 h	2 weeks	14 h
Riemersma-van der Lek (2008) [39]	Full-spectrum white light	LED, NR	Light tubes	NR	1000 lux	10:00–18:00	8 h	6 weeks	336 h
Livingston (2019) [38]	Full-spectrum white light	LED, NR	Light box	25 cm in front of patient	10000 lux	Same time every morning	0.5 h	12 weeks	42 h
Sloane (2015) [40]	Blue-enriched white light	LED, 470 nm	Table, floor lamps, and light box	NR	300–400 lux	Awakening time to 18:00	Approximately 12 h	6 weeks	504 h

NR = Not reported, ^a^ = Average lux of all three light devices used, LED = light-emitting diode, NIR = near-infrared, BLT = bright light therapy, IR = infrared, PBM = photobiomodulation.

### Evaluation of evidence quality

**Fig 2:**
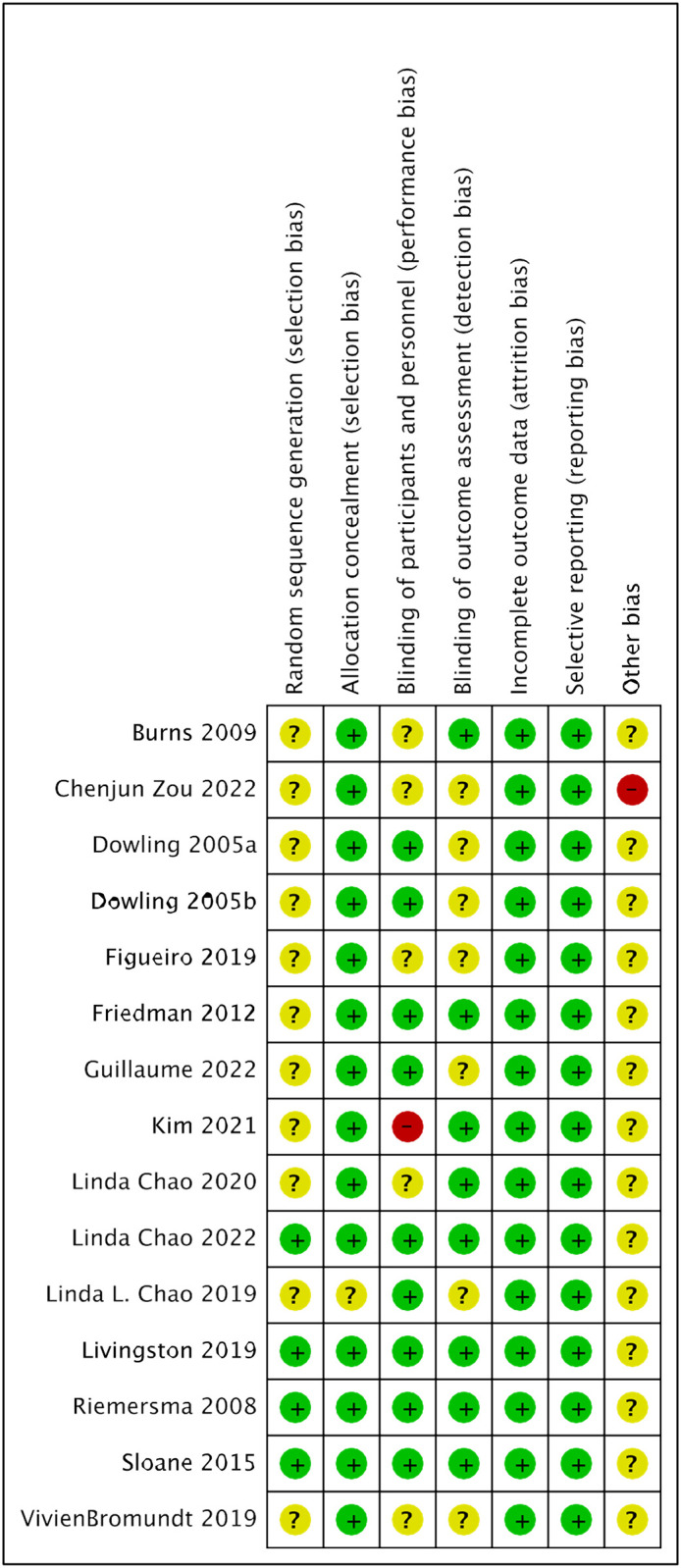
Summary of risk of bias of all included studies.

The risk of bias for each included study is presented in Fig 2. Four studies reported using random-sequence generation methods [36,38–40], while others were considered unclear risk due to insufficient information. Most studies provided details of the allocation concealment method appropriately, whereas only one [37] was assessed as unclear bias risk. Furthermore, nine studies [31,32,34,36–41] reported the blinding of participants and research personnel. One study [35] was deemed high risk of bias because it used a single-blind approach, while others had unclear risk because of insufficient information. Additionally, eight studies [28,29,35,36,38–41] described the blinding of outcome assessment, while the remaining were designated as uncertain bias risk. All articles reported complete outcomes and were consequently rated low risk for this aspect. In the case of selective reporting, all studies were judged as having low risk of bias. Finally, only one article [30] was considered high bias risk because the equipment used for treatment was provided by the hospital, while others were regarded as unclear risk owing to insufficient evidence.

### Meta analysis results

#### SE

**Fig 3:**
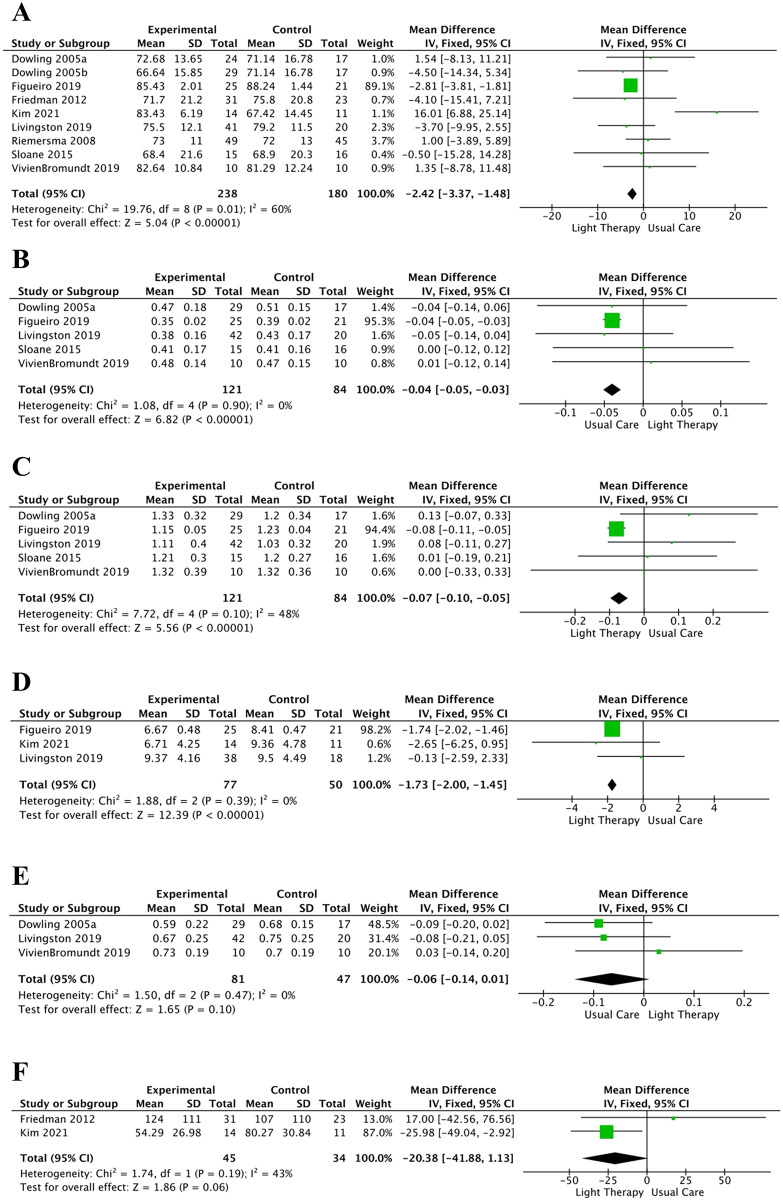
Meta-analysis of light therapy versus usual care.

SE is the total sleep time divided by the average time spent in bed, with higher SE indicating better sleep quality. Nine studies [31–33,35,38–42] reported on SE (Fig 3A), and their pooled results indicated that light therapy had a significant effect in improving SE than usual care (MD = −2.42, 95% CI = −3.37 to −1.48, *I*^*2*^ = 60%, *p* < 0.00001). Subgroup analyses of SE and the NPI are shown in Table 3.

**Table 3:** Subgroup analysis of SE and NPI.

Outcome measure	Subgroups	Studies, n	Participants, n	*I*^2^ (%)	Q-Test	Mean Difference	95% CI	*P* value
SE	**Setting**							
	*Community*	4	171	78	0.004	1.26	[−3.21, 5.74]	0.58
	*Institution*	5	247	0	0.45	−2.6	[−3.56, −1.63]	*<0*.*00001*
	**Dementia level**							
	Mild to moderate	5	239	79	0.0009	−2.42	[−3.39, −1.46]	*<0*.*00001*
	Moderate to severe	4	179	0	0.79	−2.48	[−6.90, 1.94]	0.27
	**Intervention frequency**							
	<7/w	2	87	0	0.39	−1.43	[−8.32, 5.47]	0.69
	7/w	7	331	68	0.004	−2.44	[−3.40, −1.49]	*<0*.*00001*
NPI	**Setting**							
	*Community*	3	78	57	0.1	−0.71	[−2.36, 0.94]	0.4
	Institution	2	69	14	0.28	−4.76	[−6.16, −3.36]	*<*0.00001
	**Dementia level**							
	Mild to moderate	3	32	83	0.002	−1.56	[−3.09, −0.02]	0.05
	Moderate to severe	2	115	0	0.82	−4.49	[−5.98, −3.00]	*<*0.00001
	**Intervention frequency**							
	<7/w	3	32	83	0.002	−1.56	[−3.09, −0.02]	0.05
	7/w	2	115	0	0.82	−4.49	[−5.98, −3.00]	*<*0.00001

CI = confidence interval.

#### IS

IS is used to evaluate the synchronization of the 24-hour biorhythmic patterns. In IS measurement, the theoretical range is defined as 0–1, wherein the closer the value is to 1, the greater the circadian stability. IS was mentioned in five studies [31,33,38,40,42] (Fig 3B), and our meta-analysis findings showed that light therapy could reduce daytime sleep time and avert circadian rhythm disorders in patients with AD (MD = −0.04, 95% CI = −0.05 to −0.03, *I*^*2*^ = 0%, *p* < 0.00001).

#### IV

IV is employed to quantify the fragmentation of the rest and activity cycle, with values ranging from 0 to 2. A high IV implies daytime napping and/or frequent nighttime arousals, indicating a more fragmented rhythm. IV was evaluated in five studies [31,33,38,40,42] (Fig 3C), and our meta-analysis demonstrated that light therapy could increase night sleep time and alleviate the sleep quality of patients with AD (MD = −0.07, 95% CI = −0.10 to −0.05, *I*^*2*^ = 48%, *p* < 0.00001).

#### PSQI

The PSQI is a scale administered to assess the sleep status of patients in the past month, where higher scores are associated with poorer sleep quality. Three studies [33,35,38] (Fig 3D) explored the effects of light therapy on sleep quality changes using the PSQI, and the observations exhibited that light therapy was more effective than usual care in improving sleep quality in patients with AD (MD = −1.73, 95% CI = −2.00 to −1.45, *I*^*2*^ = 0%, *p* < 0.00001).

#### RA

RA is an index designed to determine the relative difference between the average of 10 h of the highest exercise and the average of 5 h of the lowest exercise. In this index, the theoretical range is from 0 to 1, and higher values are linked with a stronger rhythm. Three articles [31,38,42] (Fig 3E) reported no significant differences between light therapy and usual care in ameliorating the RA values (MD = −0.06, 95% CI = −0.14 to 0.01, *p* = 0.10).

#### WASO

WASO is a specific parameter used to assess sleep quality, and it refers to the time spent awake between first sleep onset and awakening times. A lower WASO score is indicative of better sleep quality. Two articles [35,41] (Fig 3F) applied WASO to evaluate the effects of light therapy. The findings of our meta-analysis revealed that light therapy did not significantly enhance sleep quality in terms of WASO scores (MD = −20.38, 95% CI = −41.88 to 1.13, *p* = 0.06).

#### ADAS-cog

**Fig 4:**
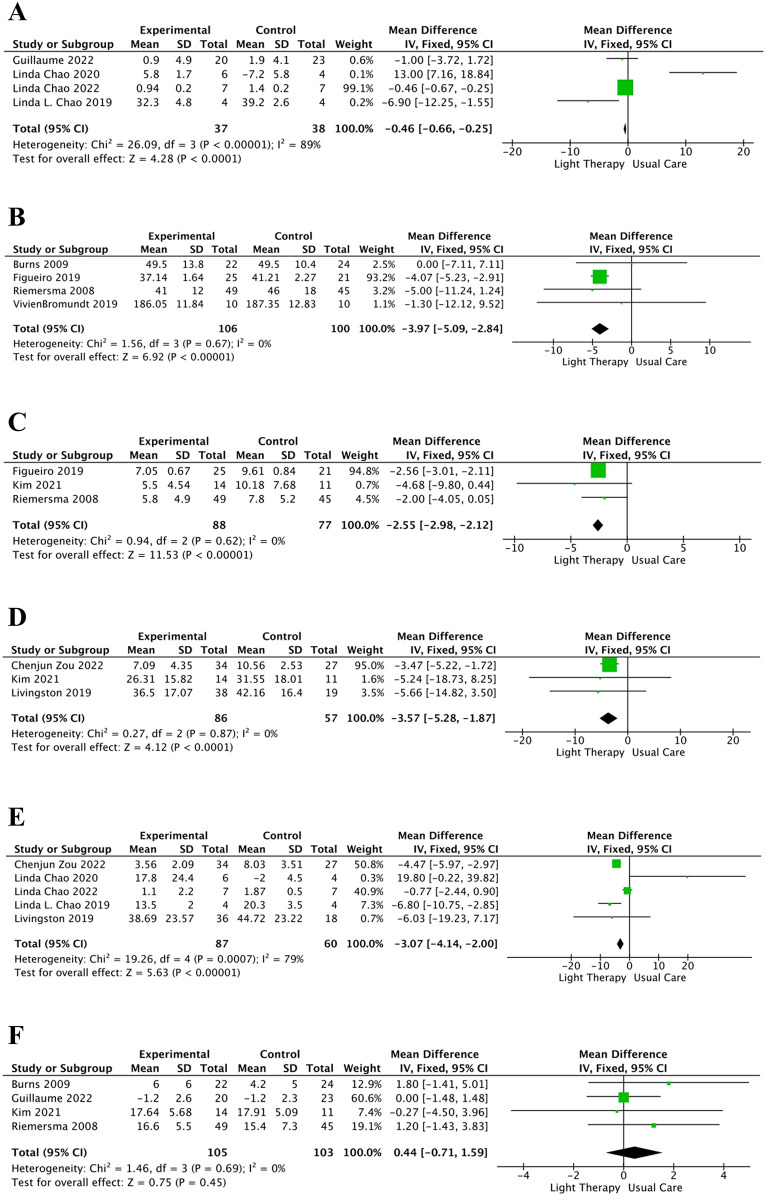
Meta-analysis of light therapy versus usual care.

The ADAS-cog is an instrument used to measure the severity of cognitive dysfunction. The ADAS-cog score spans from 0 to 70, wherein higher scores imply increased cognitive impairment. Four articles [28,34,36,37] were found to analyze the effects of light therapy on cognitive impairment via the ADAS-cog (Fig 4A). Our meta-analysis demonstrated that light therapy reduced the ADAS-cog scores and improved cognitive function in patients with AD (MD = −0.46, 95% CI = −0.66 to 0.25, *I*^*2*^ = 89%, *p* < 0.00001).

#### CMAI

The CMAI is a questionnaire constructed to gauge agitation, with scores ranging from 29 to 203. In the CMAI, lower scores indicate improved agitated behavior, while higher scores suggest more severe agitation. Four articles [29,33,39,42] utilized the CMAI score as an outcome measure (Fig 4B), and their meta-analysis demonstrated that light therapy was relatively more beneficial for improving agitated behavior in patients with AD (MD = −3.97, 95% CI = −5.09 to 2.84, *I*^*2*^ = 0%, *p* < 0.00001).

#### CSDD

The CSDD is used to assess the severity of depression-related symptoms within the last week. In this instrument, higher scores are suggestive of greater depression severity. The CSDD was applied in three studies [33,35,39] (Fig 4C), and their meta-analysis findings revealed that light therapy could alleviate depressive symptoms in patients with AD (MD = −2.55, 95% CI = −2.98 to −2.12, *I*^*2*^ = 0%, *p* < 0.00001).

#### ZBI

The ZBI is a commonly used measure of the adverse health outcomes experienced by caregivers of individuals with debilitating conditions, wherein higher scores imply higher caregiver burden. Three studies [30,35,38] examined the effect of light therapy on regulating caregiver burden using the ZBI (Fig 4D). Our meta-analysis results indicated that compared to usual care, light therapy significantly reduced caregiver burden (MD = −3.57, 95% CI = −5.28 to −1.87, *I*^*2*^ = 0%, *p* < 0.00001).

#### NPI

The NPI is a reliable assessment of the behavioral and psychological symptoms in patients with AD, with higher scores corresponding to more severe symptoms. Five articles [28,30,36–38] used the NPI to report on the effects of light therapy in patients with AD (Fig 4E). Data analysis exhibited a significant advantage of light therapy over usual care in reducing the severity of the patients’ psychobehavioral symptoms (MD = −3.07, 95% CI = −4.14 to −2.00, *p* < 0.00001, *I*^*2*^ = 79%). Subgroup analyses of SE and the NPI are shown in Table 3.

#### MMSE

The MMSE is a commonly administered screening tool to assess cognitive and intellectual decline in older adults, with lower scores reflecting more severe cognitive impairment. Four studies [29,34,35,39] examined the effect of light therapy on the changes in the MMSE scores (Fig 4F). Our meta-analysis exhibited no statistical significance in the effect of light therapy on improving cognition in patients with AD (MD = 0.44, 95% CI = −0.71 to 1.59, *p* = 0.45).

## Discussion

AD is a degenerative disorder of the central nervous system characterized by progressive cognitive decline and impaired memory function. This neurodegenerative disease is the fourth leading cause of mortality among older adults, below cardiovascular disease, cancer, and stroke [43]. Moreover, the insidious onset, long disease course, irreversible brain injury, and difficulty in patient care associated with AD have made it a public health and social concern requiring urgent resolution [44]. Although vast research has been conducted in pharmacological treatment for AD, the current pharmacological treatments can only reduce clinical symptoms to a certain extent; however, they cannot stop or reverse the pathological process. Further, AD medication carries side effects, such as diarrhea, muscle cramps, weakness, nausea, vomiting, and insomnia, notably reducing patient compliance with such drugs [45,46]. Therefore, physical interventions with relative safety, low cost, and fewer side effects are more likely to be favored by most patients. Given these modality requirements, PBM, with its advantages of a non-invasive treatment mechanism and no side effects, can be used as an adjunct to current effective treatment and even be developed into a preventive physiotherapy intervention. In this systematic review, 15 RCTs were included for meta-analysis to establish whether PBM can improve sleep disorders and psychobehavioural symptoms in patients with AD. Overall, the results revealed that light therapy helped to alleviate SE, sleep quality, depressed mood, caregiver burden, and agitated behavior. Additionally, we conducted a subgroup analysis of the indicators of high heterogeneity. The possible sources of heterogeneity were explored via subgroup analysis; however, the results demonstrated that the environment, the degree of dementia, and frequency were not heterogeneity sources. This observation may have been due to the limited number of included research investigations, indicating the requirement to constantly expand the study sample size and perform multi-center clinical research in the future to provide feasible solutions for promoting the application of light therapy.

### SE and PSQI

Ambient light information is conveyed by melanopsin-expressing retinal ganglion cells (mRGCs) via the retinohypothalamic tract that projects to the SCN of the brain’s hypothalamus [47]. The sleep quality in patients with AD can be improved by synchronizing the central biological clock with external light rhythms [48]. In this systematic review, a few studies applied the PSQI and SE scores in their research and showed that light therapy could enhance sleep quality in patients with AD, consistent with the meta-analysis results of Chiu et al. [49] and Maanen et al. [21]. Although our meta-analysis suggested that light therapy could alleviate sleep quality in patients having AD, the results should be interpreted cautiously due to the general inconsistency in the calculation of SE [50]. Thus, additional investigations are necessary to confirm these results.

### CSDD

In mammals, light is perceived by the rods and cones as well as by a subset of retinal ganglion cells that express the photopigment melanopsin that renders them intrinsically photosensitive (ipRGCs) [51]. These ipRGCs adjust the human circadian clock to the environment, regulate the sleep-wake cycle, and modulate mood [52,53]. Furthermore, light is crucial in improving neurobehavioral performance via melatonin secretion regulation, a likely mechanism by which light exerts its antidepressant effect [54]. Correspondingly, researchers have investigated the use of light therapy in psychiatry since the 1980s. Kripke et al. [55] were the first to discover that light can ameliorate depressive symptoms in patients with seasonal affective disorder. A meta-analysis by Golden et al. [56] further confirmed the effectiveness of light therapy in treating this condition. However, the efficacy of light therapy for non-seasonal depression remains disputed, possibly due to the inconsistencies in the light intensity and study population across various studies. Our present systematic review revealed that light therapy improved depression in patients with AD, generally congruent with the results of Tao et al. [57]. Despite these supporting results, whether disease severity has an influence on the efficacy of light therapy interventions remains uncertain due to the limited number of studies involving patients with mild and severe AD. Considering this shortcoming, future studies should focus on validating the effect of light therapy on depressive mood in patients with AD.

### CMAI

One of the most prevalent behavioral symptoms in patients with AD is agitation, usually manifesting as abnormal vocalization, restlessness, and repetitive movement, along with certain psychological burdens and safety risks to patients and their caregivers. Our meta-analysis showed that light therapy significantly reduced aggressive behavior in patients having AD. We speculate that light therapy may reduce agitated behavior in these patients by regulating their circadian rhythm and improving sleep quality. However, this finding differed from that of Barrick et al. [58], where no beneficial effect of bright light on agitation was detected. Several possible reasons could have caused the inconsistent result. Most patients in our included studies were those with mild to moderate AD, with only one research involving those with moderate to severe AD. Thus, these patient populations were distinct from the included population in the study by Barrick et al. [58], which demonstrated that participants with severe AD were slightly more agitated during light exposure. Finally, variation in light intensity is also a major cause of inconsistent results. In our included studies, a light intensity of <2500 lux was used, contrasting the experimental conditions employed by Barrick et al. Moreover, the relationship between light therapy and agitation in patients with AD has been debated for over a decade [59]. Overall, the results of light therapy for agitated behavior are mixed, mainly due to the lack of consensus on the intensity of illumination, duration, and daily exposure, warranting further investigation on light therapy for treating agitation in patients with AD.

### IS and IV

The mammalian biological clock consists of central and peripheral clocks. Among them, the central biological clock is centered around the SCN [60], from which light signals are projected to the brain’s pineal gland. This tiny endocrine gland regulates melatonin secretion [61], manages the body’s physiological time, and coordinates and controls the body’s rhythm and activity status during different times of the day. The synchrony of the 24-hour biorhythmic patterns was evaluated using IS, while the fragmentation of the circadian rhythms was assessed via IV. Moreover, studies have found that patients with AD have poor daytime stability and greater diurnal variability [62]. Our meta-analysis results showed that phototherapy corrected the sleep and rest times of patients with AD and stabilized their circadian rhythms, facilitating the development of good sleep habits. These findings were supported by the results of Van Someren et al. [17].

### RA

The current meta-analysis revealed no statistically significant observations for the effect of light therapy on the RA of circadian rhythms. A higher RA score suggests that patients are more active during the day and less at night, implying better sleep quality. The lack of statistical significance in the RA measures may be ascribed to the predominantly female study population (female:male = 60:48) in the included literature. Studies have reported [21] certain gender differences between the responses of men and women to light, with men exhibiting more light sensitivity than women [63]. The effect of gender on all other measures requires further confirmation by expanding the sample size.

### MMSE and ADAS-cog

AD is a degenerative neurological disorder characterized by greater severity in cognitive decline than during normal aging. Moreover, growing evidence supports the link between circadian rhythm disturbances and cognitive dysfunction in the AD course. Circadian rhythm and sleep-wake alterations often occur early in the disease course and accelerate the progression of cognitive impairment in patients with AD [64]. In this systematic review, certain studies used the MMSE and ADAS-cog to assess changes in cognitive function. Our meta-analysis results indicated that light therapy reduced the ADAS-cog scores but did not significantly affect the MMSE measures. Nevertheless, we cannot rule out the possibility of result variability due to patient population differences. Specifically, ADAS-cog included trials in which all participants were diagnosed with AD, whereas in MMSE, only the majority of participants (70%) were, the remaining participants had other types of dementia. Thus, light therapy may affect patients with distinct types of dementia differently. Hence, investigators should consider the importance of factoring in these pathology differences when designing studies on light therapy.

### NPI and ZBI

The insidious onset, long course, and progressive development of AD frequently result in cognitive impairment and personality changes, as well as place a heavy burden on the family and society that can even seriously affect the quality of life of the patient’s immediate family [65]. Moreover, in the context of caregiver burden, our meta-analysis found that the ZBI scores were reduced after light therapy. We also found that the NPI scores were significantly lower following light therapy than after usual care, in line with the results of Dowling et al. [66]. However, the inference from these outcome indicators should be cautiously assessed due to the limited number of patients in the included studies.

### WASO

Our meta-analysis indicated that light therapy had no significant effect on WASO, contrasting the findings of Li et al. [67] An explanation for the incongruent results could be the difference in the inclusion populations. The population enrolled in the studies in our review consisted of patients with AD, whereas the population investigated by Li et al. comprised those with prominent sleep disorders. Furthermore, the interventions could have been insufficiently personalized because they were commonly applied across institutions.

Overall, our current systematic review suggested that light therapy is more effective than usual care. However, most included studies involve small sample sizes, while no agreement on light intensity and intervention duration currently exists. Thus, the sample size of studies should be continually expanded and multicenter clinical studies are required to determine the optimal treatment modality that can be administered. Another meta-analysis showed [68] that bright light in an environment could increase depressive and anxious behaviors in patients because it may be considered unnatural and lead to uncomfortableness due to its relative invasiveness, causing adverse behavioral outcomes. Therefore, light exposure should be maintained within acceptable levels. Moreover, the patterns of 24-hour melatonin secretion were detected to be irregular in patients with AD. Hence, in light therapy protocols for AD, the light exposure duration should be customized to the individual’s circadian rhythm to maximize efficacy [69]. Furthermore, light therapy studies should be conducted progressively on different types of sleep disorders in patients with AD to benefit these varied patient populations. Lastly, portable light therapy devices for AD are required, presenting a promising new direction in the clinical management of AD and a viable solution to alleviate the burden of patient care and medical treatment associated with this neurodegenerative disease.

### Limitations

Our meta-analysis has limitations that are worth considering. The types and degrees of dementia included in our meta-analysis investigation were inconsistent, potentially affecting the outcome indicators. Additionally, a few articles did not clearly describe their randomization and allocation concealment methods, indicating possible bias in these studies.

## Conclusion

Our systematic review and meta-analysis revealed that light therapy significantly improved sleep and psychobehavioral symptoms in patients with AD. These findings combined with its low side effects suggest the role of light therapy as a promising treatment for AD. Although light therapy has fewer side effects than pharmacological treatment, adverse behavioral outcomes in patients due to bright light exposure should be considered. Nevertheless, further studies with appropriately larger sample sizes are necessitated to elucidate the effectiveness of light therapy in treating sleep disorders and psychobehavioral symptoms in patients with AD.

## Supporting information

S1 Fig The funnel plots. (TIF)

S1 Table The PRISMA checklist. (DOCX)

S2 Table The list of keywords used for literature search. (DOCX)

S3 Table The Minimal data set. (DOCX)
