# Supplementary figures and images for: The effect of light therapy on sleep disorders and psychobehavioral symptoms in patients with Alzheimer’s disease: A meta-analysis
Source: PLoS One. 2023 Dec 6;18(12):e0293977. doi: 10.1371/journal.pone.0293977 (PMC10699648; doi:10.1371/journal.pone.0293977)

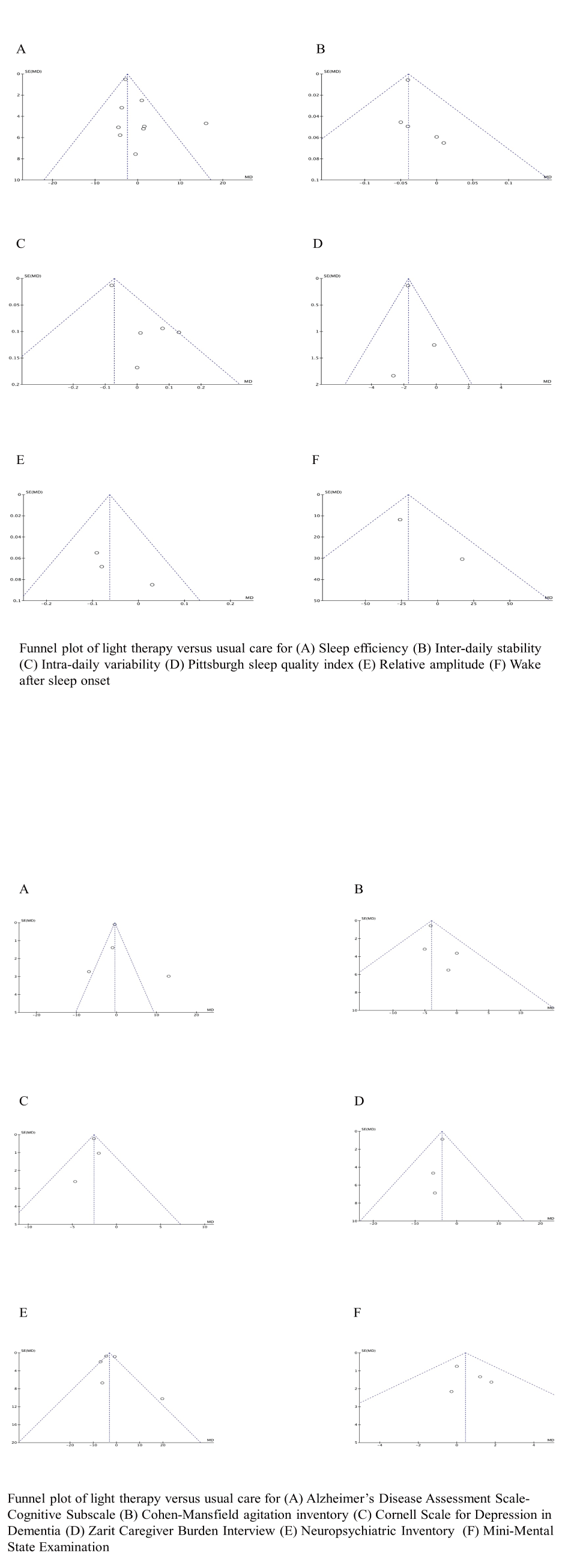

Supplement: S1 Fig — (TIF) [file pone.0293977.s001.tif]
