# Supplementary material for: The effect of light therapy on sleep disorders and psychobehavioral symptoms in patients with Alzheimer’s disease: A meta-analysis
Source: PLoS One. 2023 Dec 6;18(12):e0293977. doi: 10.1371/journal.pone.0293977 (PMC10699648; doi:10.1371/journal.pone.0293977)
Supplement: S2 Table — (DOCX) [file pone.0293977.s003.docx]

The effect of light therapy on sleep disorders and psychobehavioral symptoms in patients with Alzheimer's disease: A meta-analysis

Supporting Information

List of Keywords for Literature Search

***Search-String Alzheimer Disease***

"Alzheimer Dementia" OR "Alzheimer Dementias" OR "Dementia, Alzheimer" OR "Alzheimer's Disease" OR "Dementia, Senile" OR "Senile Dementia" OR "Dementia, Alzheimer Type" OR "Alzheimer Type Dementia" OR "Alzheimer-Type Dementia" OR "Dementia, Alzheimer-Type" OR "Alzheimer Type Senile Dementia" OR "Primary Senile Degenerative Dementia" OR "Dementia, Primary Senile Degenerative" OR "Alzheimer Sclerosis" OR "Sclerosis, Alzheimer" OR "Alzheimer Syndrome" OR "Alzheimer's Diseases" OR "Alzheimer Diseases" OR "Alzheimers Diseases" OR "Senile Dementia, Alzheimer Type" OR "Acute Confusional Senile Dementia" OR "Senile Dementia, Acute Confusional" OR "Dementia, Presenile" OR "Presenile Dementia" OR "Alzheimer Disease, Late Onset" OR "Late Onset Alzheimer Disease" OR "Alzheimer's Disease, Focal Onset" OR "Focal Onset Alzheimer's Disease" OR "Familial Alzheimer Disease" OR "Alzheimer Disease, Familial" OR "Familial Alzheimer Diseases" OR "Alzheimer Disease, Early Onset" OR "Early Onset Alzheimer Disease" OR "Presenile Alzheimer Dementia"

***Search-String Phototherapy***

"Phototherapies" OR "Therapy, Photoradiation" OR "Photoradiation Therapies" OR "Therapies, Photoradiation" OR "Light Therapy" OR "Light Therapies" OR "Therapies, Light" OR "Therapy, Light" OR "Photoradiation Therapy" OR "BLT" OR "Bright Light Therapy" OR "Bright Light"

***Search-String Sleep Disorders, Circadian Rhythm***

"Sleep-Wake Schedule Disorders" OR "Sleep Wake Schedule Disorders" OR "Sleep-Wake Schedule Disorder" OR "Sleep Wake Schedule Disorder" OR "Circadian Rhythm Sleep Disorders" OR "Disturbed Nyctohemeral Rhythm" OR "Disturbed Nyctohemeral Rhythms" OR "Nyctohemeral Rhythm, Disturbed" OR "Nyctohemeral Rhythms, Disturbed" OR "Sleep-Wake Cycle Disorder" OR "Sleep Wake Cycle Disorder" OR "Circadian Rhythm Sleep Disorder" OR "Sleep-Wake Cycle Disorders" OR "Sleep Wake Cycle Disorders" OR "Shift-Work Sleep Disorder" OR "Shift Work Sleep Disorder" OR "Sleep Disorder, Shift-Work" OR "Shift-Work Sleep Disorders" OR "Sleep Disorder, Shift Work" OR "Sleep Disorders, Shift-Work" OR "Non-24 Hour Sleep-Wake Disorder" OR "Non 24 Hour Sleep Wake Disorder" OR "Sleep-Wake Disorder, Non-24 Hour" OR "Sleep Wake Disorder, Non 24 Hour" OR "Nonorganic Sleep Wake Cycle Disorders" OR "Nonorganic Sleep Wake Cycle Disorder" OR "Advanced Sleep Phase Syndrome" OR "Delayed Sleep Phase Syndrome" OR "Delayed Sleep-Phase Syndrome" OR "Delayed Sleep-Phase Syndromes"

# ***Search-String Cognition Disorders***

"Disorder, Cognition" OR "Disorders, Cognition" OR "Overinclusion"

We searched the databases Embase, the Clinical Trials Registry, Web of science, PubMed and the Cochrane Library. For each of the databases we ran the above keyword search with separate title, keywords, and abstract filters for each search-string.
