# Supplementary material for: The effect of light therapy on sleep disorders and psychobehavioral symptoms in patients with Alzheimer’s disease: A meta-analysis
Source: PLoS One. 2023 Dec 6;18(12):e0293977. doi: 10.1371/journal.pone.0293977 (PMC10699648; doi:10.1371/journal.pone.0293977)
Supplement: S3 Table — (DOCX) [file pone.0293977.s004.docx]

The effect of light therapy on sleep disorders and psychobehavioral symptoms in patients with Alzheimer's disease: A meta-analysis

Supporting Information

Minimal data set

***Study Title: A Randomized, Double-Blind, and Sham-Controlled Trial of an Innovative Brain-Gut Photobiomodulation Therapy:Safety and Patient Compliance***

Characteristics of Research Subjects

| First author | Guillaume |
| --- | --- |
| Year of publication | 2022 |
| Publication type | Journal |
| Number of patients (Intervention/Control) | 53 (27/26) |
| Age (Mean ± SD) | Intervention: 72.4 ± 7.0  Control: 73.7 ± 6.4 |
| Gender (Male/Female) | Intervention:: 12/15  Control: 10/16 |
| Country | France |
| Measures for the intervention group | All patients had 40 treatment sessions lasting 25 min each over 8 weeks and were followed for 4 weeks afterwards |
| Measures for the control group | All NIR emissions were inactivated |
| Setting | Nursing Home |
| Light therapy | Intervention: PBM medical device  Control: All NIR emissions were inactivated |
| MMSE score | 20.5 ± 3.6 |
| Type of light therapy | Laser diode, LED |
| Light source / wave length | Laser diode: 850 nm  IR LED: 850 nm  Red LED: 660 nm |
| Light therapy device | PBM medical device RGn530 |
| Distance from light to patient | NR |
| Light intensity | NR |
| Intervention time | NR |
| Total time exposed to light therapy | Approximately 17 h |
| Follow-up time | Followed for a 4−week period after treatment discontinuation |
| Outcomes | ADAS-cog, MMSE |

| Continuous data  (pre-intervention) | Intervention | | | Control | | |
| --- | --- | --- | --- | --- | --- | --- |
|  | N | Means | SD | N | Means | SD |
| MMSE | 27 | 20.5 | 3.6 | 26 | 20.2 | 3.5 |
| ADAS-cog | 27 | 20.4 | 6.9 | 26 | 23.1 | 8.6 |
|  |  |  |  |  |  |  |
| Continuous data  (post-intervention) | Intervention | | | Control | | |
|  | N | Means | SD | N | Means | SD |
| MMSE | 20 | -1.2 | 2.6 | 23 | -1.2 | 2.3 |
| ADAS-cog | 20 | 0.9 | 4.9 | 23 | 1.9 | 4.1 |

***Study Title: A Randomized, Double-Blind, and Sham-Controlled Trial of an Innovative Brain-Gut Photobiomodulation Therapy:Safety and Patient Compliance***

Characteristics of Research Subjects

| First author | Dowling 2005a |
| --- | --- |
| Year of publication | 2005 |
| Publication type | Journal |
| Number of patients (Intervention/Control) | 46 (29/17) |
| Age (Mean ± SD) | 84 ± 10 |
| Gender (Male/Female) | 36/10 |
| Country | USA |
| Measures for the intervention group | The experimental group received one hour (09:30−10:30) of bright light exposure (≥ 2500 lux in gaze direction) Monday through Friday for 10 weeks |
| Measures for the control group | The control group received usual indoor light (150−200 lux) |
| Setting | LTC facilities |
| Light therapy | Intervention: Bright light  Control: Usual indoor light |
| MMSE score | 7 ± 7 |
| Type of light therapy | Full spectrum white light |
| Light source / wave length | NR |
| Light therapy device | Light box |
| Distance from light to patient | 1.2 m in front of patient |
| Light intensity | ≥2500 lux |
| Intervention time | 09:30−10:30 |
| Total time exposed to light therapy | 50 h |
| Follow-up time | NR |
| Outcomes | IS, IV, SE, RA |

| Continuous data  (pre-intervention) | Intervention | | | Control | | |
| --- | --- | --- | --- | --- | --- | --- |
|  | N | Means | SD | N | Means | SD |
| SE | 29 | 63.02 | 19.27 | 17 | 66.88 | 19.24 |
| IS | 29 | 0.36 | 0.16 | 17 | 0.47 | 0.18 |
| IV | 29 | 1.27 | 0.35 | 17 | 1.17 | 0.45 |
| RA | 29 | 0.52 | 0.22 | 17 | 0.62 | 0.21 |
| Continuous data  (post-intervention) | Intervention | | | Control | | |
|  | N | Means | SD | N | Means | SD |
| SE | 29 | 66.64 | 15.58 | 17 | 71.14 | 16.78 |
| IS | 29 | 0.47 | 0.18 | 17 | 0.51 | 0.15 |
| IV | 29 | 1.33 | 0.32 | 17 | 1.20 | 0.34 |
| RA | 29 | 0.59 | 0.22 | 17 | 0.68 | 0.15 |

***Study Title: Effect of timed bright light treatment for rest-activity disruption in institutionalized patients with Alzheimer’s disease***

Characteristics of Research Subjects

| First author | Dowling 2005b |
| --- | --- |
| Year of publication | 2005 |
| Publication type | Journal |
| Number of patients (Intervention/Control) | 70 (I_1_:29, I_2_:24/17) |
| Age (Mean ± SD) | 84 ± 10 |
| Gender (Male/Female) | 13/57 |
| Country | USA |
| Measures for the intervention group | The experimental group received one hour (09:30−10:30)of bright light exposure (≥ 2500 lux in gaze direction) Monday through Friday for 10 weeks |
| Measures for the control group | The control group received usual indoor light (150−200 lux) |
| Setting | LTC facilities |
| Light therapy | I_1_: Morning bright light  I_2_: Afternoon bright light  Control: Usual indoor light |
| MMSE score | 7 ± 7 |
| Type of light therapy | Full spectrum white light |
| Light source / wave length | NR |
| Light therapy device | Light box |
| Distance from light to patient | 1.2 m in front of patient |
| Light intensity | ≥2500 lux |
| Intervention time | 15:30−16:30 |
| Total time exposed to light therapy | 50 h |
| Follow-up time | NR |
| Outcomes | SE |

| Continuous data  (pre-intervention) | Intervention | | | Control | | | | | |
| --- | --- | --- | --- | --- | --- | --- | --- | --- | --- |
|  |  |  |  | Am | | | Pm | | |
|  | N | Means | SD | N | Means | SD | N | Means | SD |
| SE | 17 | 66.88 | 19.24 | 29 | 63.02 | 19.57 | 24 | 72.7 | 13.58 |
| Continuous data  (post-intervention) | Intervention | | | Control | | | | | |
|  |  |  |  | Am | | | Pm | | |
|  | N | Means | SD | N | Means | SD | N | Means | SD |
| SE | 17 | 71.14 | 16.78 | 29 | 66.64 | 15.85 | 24 | 72.68 | 13.65 |

***Study Title: Effects of Home Photobiomodulation Treatments on Cognitive and Behavioral Function, Cerebral Perfusion, and Resting-State Functional Connectivity in Patients with Dementia: A Pilot Trial***

Characteristics of Research Subjects

| First author | Linda L.Chao |
| --- | --- |
| Year of publication | 2019 |
| Publication type | Journal |
| Number of patients (Intervention/Control) | 8 (4/4) |
| Age (Mean ± SD) | 80.5 ± 6.5/79.0 ± 5.9 |
| Gender (Male/Female) | I: 1/3  C: 2/2 |
| Country | USA |
| Measures for the intervention group | Received PBM at home 3 days per week for 12 weeks. |
| Measures for the control group | 12 weeks of daily activities, usual care |
| Setting | LTC facilities |
| Light therapy | Intervention: Home PBM treatments  Control: Usual care |
| MMSE score | 19.5 ± 7 |
| Type of light therapy | NIR |
| Light source / wave length | LED, 810 nm |
| Light therapy device | Transcranial and intranasal LEDs of a PBM device |
| Distance from light to patient | NR |
| Light intensity | NR |
| Intervention time | NR |
| Total time exposed to light therapy | 12 h |
| Follow-up time | NR |
| Outcomes | ADAS-cog, NPI |

| Continuous data  (pre-intervention) | Intervention | | | | | | Control | | | | | | | |
| --- | --- | --- | --- | --- | --- | --- | --- | --- | --- | --- | --- | --- | --- | --- |
|  | N | | Means | SD | | | N | | Means | | | SD | | |
| ADAS-cog | 4 | | 37.5 | 5.5 | | | 4 | | 32.1 | | | 0.3 | | |
| NPI | 4 | | 35.0 | 11.6 | | | 4 | | 10.5 | | | 1.8 | | |
| Continuous data  (post-intervention) | Intervention | | | | | | Control | | | | | | | |
|  | 6week | | | 12week | | | 6week | | | | 12week | | | |
|  | N | Means | SD | N | Means | SD | N | Means | | SD | N | | Means | SD |
| ADAS-cog | 4 | 35.7 | 4.7 | 4 | 32.3 | 4.8 | 4 | 34.8 | | 1.2 | 4 | | 39.2 | 2.6 |
| NPI | 4 | 22.8 | 4.0 | 4 | 13.5 | 2.0 | 4 | 14.5 | | 3.1 | 4 | | 20.3 | 3.5 |

***Study Title：Brief morning light treatment for sleep/wake disturbances in older memory- impaired individuals and their caregivers***

Characteristics of Research Subjects

| First author | Friedman |
| --- | --- |
| Year of publication | 2012 |
| Publication type | Journal |
| Number of patients (Intervention/Control) | 54 (31/23) |
| Age (Mean ± SD) | 77.9 ± 8.1 |
| Gender (Male/Female) | I: 20/11  C: 13/10 |
| Country | USA |
| Measures for the intervention group | AD participants received 30 min of bright light within 30 min of waking up each day for 2 weeks |
| Measures for the control group | AD participants were exposed to 30 min of dim light within 30 min of waking up each day for 2 weeks |
| Setting | Community |
| Light therapy | Intervention: Morning bright light  Control: Dim red light |
| MMSE score | 22.1 ± 4.7 |
| Type of light therapy | Full spectrum white light |
| Light source / wave length | NR |
| Light therapy device | Light box |
| Distance from light to patient | NR |
| Light intensity | 4200 lux |
| Intervention time | Initiated within 30 min of patient's wake time |
| Total time exposed to light therapy | 7 h |
| Follow-up time | NR |
| Outcomes | WASO, SE |

| Continuous data  (pre-intervention) | Intervention | | | Control | | |
| --- | --- | --- | --- | --- | --- | --- |
|  | N | Means | SD | N | Means | SD |
| WASO | 31 | 124 | 98.1 | 23 | 108 | 102 |
| SE | 31 | 72.9 | 17 | 23 | 75.1 | 17.8 |
| Continuous data  (post-intervention) | Intervention | | | Control | | |
|  | N | Means | SD | N | Means | SD |
| WASO | 31 | 124 | 111 | 23 | 107 | 110 |
| SE | 31 | 71.7 | 21.2 | 23 | 75.8 | 20.8 |

***Study Title: Photobiomodulation for Improving Brain Function in Dementia (PBM Dementia) (PBM Dementia)***

Characteristics of Research Subjects

| First author | Linda Chao |
| --- | --- |
| Year of publication | 2020 |
| Publication type | the Clinical Trials Registry |
| Number of patients (Intervention/Control) | 10 (6/4) |
| Age (Mean ± SD) | 78.3 ± 10.2/75.0 ± 10.8 |
| Gender (Male/Female) | I: 3/3 C: 2/2 |
| Country | USA |
| Measures for the intervention group | Receive photobiomodulation (PBM) provided with the Vielight Gamma device every other day (e.g., Monday, Wednesday, Friday) for 20 min (the device automatically shuts off after 20 min) for 12 weeks |
| Measures for the control group | Delayed PBM treatment |
| Setting | Community |
| Light therapy | Intervention: Immediate PBM treatment  Control: Delayed PBM treatment |
| MMSE score | 19.5 ± 7 |
| Type of light therapy | NIR |
| Light source / wave length | NR |
| Light therapy device | Transcranial and intranasal NIR light of a PBM device |
| Distance from light to patient | NR |
| Light intensity | NR |
| Intervention time | NR |
| Total time exposed to light therapy | 16 h |
| Follow-up time | NR |
| Outcomes | ADAS-cog, NPI |

| Continuous data  (post-intervention) | Intervention | | | Control | | |
| --- | --- | --- | --- | --- | --- | --- |
|  | N | Means | SD | N | Means | SD |
| ADAS-cog | 6 | 5.8 | 1.7 | 4 | -7.2 | 5.8 |
| NPI | 6 | 17.8 | 24.4 | 4 | -2 | 4.5 |

***Study Title: Impact of Photobiomodulation (PBM) on Biomarkers of Alzheimer's Disease (PBMbiomarker)***

Characteristics of Research Subjects

| First author | Linda Chao |
| --- | --- |
| Year of publication | 2022 |
| Publication type | the Clinical Trials Registry |
| Number of patients (Intervention/Control) | 14 (7/7) |
| Age (Mean ± SD) | 68.1/72.4 |
| Gender (Male/Female) | I: 3/4  C: 3/4 |
| Country | USA |
| Measures for the intervention group | Transcranial and intranasal NIR light  (Monday, Wednesday, Friday) for 20 min for 16 weeks |
| Measures for the control group | No stimulation |
| Setting | Community |
| Light therapy | Intervention: Transcranial and intranasal NIR light  Control: No stimulation |
| MMSE score | 23.4 ± 7.1 |
| Type of light therapy | NIR |
| Light source / wave length | NR |
| Light therapy device | Transcranial and intranasal NIR light of a PBM device |
| Distance from light to patient | NR |
| Light intensity | NR |
| Intervention time | NR |
| Total time exposed to light therapy | Approximately 21 h |
| Follow-up time | NR |
| Outcomes | ADAS-cog, NPI |

| Continuous data  (pre-intervention) | Intervention | | | Control | | |
| --- | --- | --- | --- | --- | --- | --- |
|  | N | Means | SD | N | Means | SD |
| ADAS-cog | 7 | 35.5 | 8.3 | 7 | 27.5 | 20 |
| NPI | 7 | 42 | 29.8 | 7 | 21 | 50.7 |
| Continuous data  (post-intervention) | Intervention | | | Control | | |
|  | N | Means | SD | N | Means | SD |
| ADAS-cog | 7 | 0.94 | 0.2 | 7 | 1.4 | 0.2 |
| NPI | 7 | 1.1 | 2.2 | 7 | 1.87 | 0.5 |

***Study Title: Effect of light therapy on delirium in older patients with Alzheimer’s disease-related dementia***

Characteristics of Research Subjects

| First author | Chenjun Zou |
| --- | --- |
| Year of publication | 2022 |
| Publication type | Journal |
| Number of patients (Intervention/Control) | 61 (34/27) |
| Age (Mean ± SD) | 75.94 ± 9.47/73.04 ± 9.34 |
| Gender (Male/Female) | I: 16/18  C: 12/15 |
| Country | China |
| Measures for the intervention group | The lights are on from 9 a.m. to 9:30 a.m. The peak is 14,000 lux for 4 weeks |
| Measures for the control group | Exposed to 50 lux dim light |
| Setting | Nursing home |
| Light therapy | Intervention: Light therapy  Control: Dim light |
| MMSE score | 9.18 ± 4.56 |
| Type of light therapy | Full spectrum white light |
| Light source / wave length | NR |
| Light therapy device | Light box |
| Distance from light to patient | Within 50 cm |
| Light intensity | 1400 lux |
| Intervention time | 09:00−09:30 |
| Total time exposed to light therapy | 14 h |
| Follow-up time | NR |
| Outcomes | NPI, ZBI |

| Continuous data  (pre-intervention) | Intervention | | | | | | Control | | | | | | | |
| --- | --- | --- | --- | --- | --- | --- | --- | --- | --- | --- | --- | --- | --- | --- |
|  | N | | Means | SD | | | N | | Means | | | SD | | |
| NPI | 34 | | 41.5 | 6.96 | | | 27 | | 42.81 | | | 5.44 | | |
| ZBI | 34 | | 19.41 | 7.31 | | | 27 | | 18.7 | | | 5.2 | | |
| Continuous data  (post-intervention) | Intervention | | | | | | Control | | | | | | | |
|  | 2week | | | 4week | | | 2week | | | | 4week | | | |
|  | N | Means | SD | N | Means | SD | N | Means | | SD | N | | Means | SD |
| NPI | 34 | 12.79 | 4.58 | 34 | 3.65 | 2.09 | 27 | 18.11 | | 6.19 | 27 | | 8.03 | 3.51 |
| ZBI | 34 | 7.09 | 4.35 | 34 | 2.44 | 1.52 | 27 | 10.56 | | 2.53 | 27 | | 3.22 | 2.01 |

***Study Title: Effects of a dawn-dusk simulation on circadian rest-activity cycles, sleep, mood and well-being in dementia patients***

Characteristics of Research Subjects

| First author | Vivien Bromundt |
| --- | --- |
| Year of publication | 2019 |
| Publication type | Journal |
| Number of patients (Intervention/Control) | 20 (10/10) |
| Age (Mean ± SD) | 85.6 ± 5.8 |
| Gender (Male/Female) | 3/17 |
| Country | Switzerland |
| Measures for the intervention group | Morning bright light |
| Measures for the control group | Dim light |
| Setting | Nursing Home |
| Light therapy | Intervention: Morning bright light  Control: Dim light |
| MMSE score | 13.15 ± 10.3 |
| Type of light therapy | Full spectrum white light |
| Light source / wave length | LED, NR |
| Light therapy device | Light tubes |
| Distance from light to patient | NR |
| Light intensity | 0.35 lux−130 lux |
| Intervention time | 90 min of morning light and 90 min of afternoon light |
| Total time exposed to light therapy | 357 h |
| Follow-up time | NR |
| Outcomes | IV, IS, RA, SE, CMAI |

| Continuous data  (post-intervention) | Intervention | | | Control | | |
| --- | --- | --- | --- | --- | --- | --- |
| outcomes | N | Means | SD | N | Means | SD |
| IV | 10 | 1.32 | 0.39 | 10 | 1.32 | 0.36 |
| IS | 10 | 0.48 | 0.14 | 10 | 0.47 | 0.15 |
| RA | 10 | 0.73 | 0.19 | 10 | 0.70 | 0.19 |
| SE | 10 | 82.64 | 10.84 | 10 | 81.29 | 12.24 |
| CMAI | 10 | 186.05 | 11.84 | 10 | 187.35 | 12.83 |

***Study Title: Bright light therapy for agitation in dementia: a randomized controlled trial***

Characteristics of Research Subjects

| First author | Burns |
| --- | --- |
| Year of publication | 2009 |
| Publication type | Journal |
| Number of patients (Intervention/Control) | 48 (22/26) |
| Age (Mean ± SD) | 84.5 ± 8/82.5 ± 7.6 |
| Gender (Male/Female) | I: 6/16  C: 10/16 |
| Country | UK |
| Measures for the intervention group | Bright light therapy |
| Measures for the control group | Standard light |
| Setting | Nursing home |
| Light therapy | Intervention: Bright light therapy  Control: Standard light |
| MMSE score | 6.9 ± 5.3 |
| Type of light therapy | Full spectrum BLT |
| Light source / wave length | LED, NR |
| Light therapy device | Light box |
| Distance from light to patient | NR |
| Light intensity | 1000 lux |
| Intervention time | 10:00−12:00 |
| Total time exposed to light therapy | 28 h |
| Follow-up time | NR |
| Outcomes | CMAI, MMSE |

| Continuous data  (pre-intervention) | Intervention | | | | | | Control | | | | | | | |
| --- | --- | --- | --- | --- | --- | --- | --- | --- | --- | --- | --- | --- | --- | --- |
|  | N | | Means | SD | | | N | | Means | | | SD | | |
| CMAI | 26 | | 57.5 | 13.8 | | | 22 | | 62 | | | 18.4 | | |
| MMSE | 26 | | 5.1 | 5.6 | | | 22 | | 6.9 | | | 5.3 | | |
| Continuous data  (post-intervention) | Intervention | | | | | | Control | | | | | | | |
|  | 4week | | | 8week | | | 4week | | | | 8week | | | |
|  | N | Means | SD | N | Means | SD | N | Means | | SD | N | | Means | SD |
| CMAI | 26 | 50.9 | 15.6 | 24 | 49.5 | 10.4 | 26 | 50.9 | | 15.6 | 22 | | 49.5 | 13.8 |
| MMSE | 24 | 5 | 6 | 24 | 4.2 | 5 | 24 | 5 | | 6 | 24 | | 4.2 | 5 |

***Study Title: Effects of a Tailored Lighting Intervention on Sleep Quality, Rest–Activity, Mood,and Behavior in Older Adults With Alzheimer Disease and Related Dementias: A Randomized Clinical Trial***

Characteristics of Research Subjects

| First author | Figueiro |
| --- | --- |
| Year of publication | 2019 |
| Publication type | Journal |
| Number of patients (Intervention/Control) | 46 (43/44) |
| Age (Mean ± SD) | 85.1 ± 7.1 |
| Gender (Male/Female) | 32/14 |
| Country | USA |
| Measures for the intervention group | Whole day bright light |
| Measures for the control group | Dim light |
| Setting | LTC facilities |
| Light therapy | Intervention: Whole day bright Light  Control: Dim light |
| MMSE score | 14.7 ± 4.3 |
| Type of light therapy | Full spectrum white light |
| Light source / wave length | LED, 555 nm |
| Light therapy device | Light box, light tables and floor lights |
| Distance from light to patient | NR |
| Light intensity | 567 lux^a^ (a = Average lux of all three light devices used) |
| Intervention time | From habitual wake-time (06:00−08:00) to 18:00 |
| Total time exposed to light therapy | 280-336 h |
| Follow-up time | NR |
| Outcomes | PSQI, CSDD, CMAI, IS, IV, SE |

| Continuous data  (pre-intervention) | Intervention | | | Control | | |
| --- | --- | --- | --- | --- | --- | --- |
|  | N | Means | SD | N | Means | SD |
| PSQI | 25 | 10.3 | 0.4 | 21 | 9.8 | 0.44 |
| IS | 25 | 0.37 | 0.03 | 21 | 0.36 | 0.02 |
| IV | 25 | 1.22 | 0.05 | 21 | 1.17 | 0.05 |
| SE | 25 | 85.32 | 2.14 | 21 | 85.92 | 1.8 |
| CSDD | 25 | 10.3 | 1.02 | 21 | 10.73 | 0.88 |
| CMAI | 25 | 42.65 | 2.19 | 21 | 42.71 | 2.12 |
| Continuous data  (post-intervention) | Intervention | | | Control | | |
|  | N | Means | SD | N | Means | SD |
| PSQI | 25 | 6.67 | 0.48 | 21 | 8.41 | 0.47 |
| IS | 25 | 0.35 | 0.02 | 21 | 0.39 | 0.02 |
| IV | 25 | 1.15 | 0.05 | 21 | 1.23 | 0.04 |
| SE | 25 | 85.43 | 2.01 | 21 | 88.24 | 1.44 |
| CSDD | 25 | 7.05 | 0.67 | 21 | 9.61 | 0.84 |
| CMAI | 25 | 37.14 | 1.64 | 21 | 41.21 | 2.27 |

***Study Title: Positive effect of timed blue‑enriched white light on sleep and cognition in patients with mild and moderate Alzheimer’s disease***

Characteristics of Research Subjects

| First author | Kim |
| --- | --- |
| Year of publication | 2021 |
| Publication type | Journal |
| Number of patients (Intervention/Control) | 25 (14/11) |
| Age (Mean ± SD) | 77.36 ± 5.79/78.55 ± 7.71 |
| Gender (Male/Female) | I: 2/12  C: 5/6 |
| Country | Korea |
| Measures for the intervention group | Blue-enriched white light. Sit about 60 cm away from the small LED light box every morning for 2 weeks |
| Measures for the control group | Blue-enriched white light + blue attenuating sunglasses |
| Setting | Community |
| Light therapy | Intervention: Blue-enriched white light  Control:Blue-enriched white light + blue attenuating sunglasses |
| MMSE score | 16.6 |
| Type of light therapy | Blue enriched white light |
| Light source / wave length | LED, 400−700 nm |
| Light therapy device | Light box |
| Distance from light to patient | 61 cm in front of patient |
| Light intensity | 30 lux |
| Intervention time | 09:00−10:00 |
| Total time exposed to light therapy | 14 h |
| Follow-up time | NR |
| Outcomes | CSD, ZBI, SE, WASO, PSQI, MMSE |

| Continuous data  (pre-intervention) | Intervention | | | Control | | |
| --- | --- | --- | --- | --- | --- | --- |
|  | N | Means | SD | N | Means | SD |
| PSQI | 14 | 11.36 | 4.57 | 11 | 11.00 | 3.71 |
| SE | 14 | 78.69 | 11.75 | 11 | 70.16 | 7.28 |
| WASO | 14 | 60.30 | 35.07 | 11 | 86.89 | 26.07 |
| CSDD | 14 | 8.07 | 6.03 | 11 | 10.00 | 6.59 |
| ZBI | 14 | 30.46 | 15.68 | 11 | 36.36 | 17.00 |
| MMSE |  | 16.36 | 5.09 |  | 16.9 | 4.91 |
| Continuous data  (post-intervention) | Intervention | | | Control | | |
| outcomes | N | Means | SD | N | Means | SD |
| SE | 14 | 83.43 | 6.19 | 11 | 67.42 | 14.45 |
| WASO | 14 | 54.29 | 26.98 | 11 | 80.27 | 30.84 |
| CSDD | 14 | 5.50 | 4.54 | 11 | 10.18 | 7.68 |
| ZBI | 14 | 26.31 | 15.82 | 11 | 31.55 | 18.01 |
| MMSE | 14 | 17.64 | 5.68 | 11 | 17.91 | 5.09 |
| PSQI | 14 | 6.71 | 4.25 | 11 | 9.36 | 4.78 |

***Study Title: Effect of Bright Light and Melatonin on Cognitive and Noncognitive Function in Elderly Residents of Group Care Facilities A Randomized Controlled Tria*l**

Characteristics of Research Subjects

| First author | Riemersma-van der Lek |
| --- | --- |
| Year of publication | 2008 |
| Publication type | Journal |
| Number of patients (Intervention/Control) | 189 (I_1_:49, I_2_:49, I_3_:46/45) |
| Age (Mean ± SD) | I_1_:85 ± 6  I_2_:87 ± 6  I_3_:86 ± 5  C: 85 ± 5 |
| Gender (Male/Female) | I_1_: 4/45  I_2_: 2/47  I_3_: 8/38  C: 5/40 |
| Country | Netherlands |
| Measures for the intervention group | Bright light (The lights are on every day from about 9 a.m. to 6 p.m. The goal is an exposure of ±1000 lux, measuring the direction of the gaze before the eye) |
| Measures for the control group | Dim light + placebo drug |
| Setting | Nursing home |
| Light therapy | Intervention: I_1_: Bright light  I_2_: Bright light and melatonin  I_3_: Melatonin  Control: Dim light + placebo drug |
| MMSE score | 14.4 ± 6.6 |
| Type of light therapy | Full spectrum white light |
| Light source / wave length | LED, NR |
| Light therapy device | Light tubes |
| Distance from light to patient | NR |
| Light intensity | 1000 lux |
| Intervention time | 10:00−18:00 |
| Total time exposed to light therapy | 336 h |
| Follow-up time | NR |
| Outcomes | CSDD, CMAI, SE, MMSE |

| Continuous data  (pre-intervention) | Intervention | | | Control | | |
| --- | --- | --- | --- | --- | --- | --- |
|  | N | Means | SD | N | Means | SD |
| MMSE | 49 | 14.5 | 6.2 | 45 | 14.3 | 7.0 |
| CSDD | 49 | 7.4 | 6.9 | 45 | 7.6 | 5.1 |
| CMAI | 49 | 45 | 13 | 45 | 45 | 18 |
| SE | 49 | 70 | 16 | 45 | 76 | 13 |
| Continuous data  (post-intervention) | Intervention | | | Control | | |
|  | N | Means | SD | N | Means | SD |
| MMSE | 49 | 16.6 | 5.5 | 45 | 15.4 | 7.3 |
| CSDD | 49 | 5.8 | 4.9 | 45 | 7.8 | 5.2 |
| CMAI | 49 | 41 | 12 | 45 | 46 | 18 |
| SE | 49 | 73 | 11 | 45 | 72 | 13 |

***Study Title: DREAMS-START (Dementia RElAted Manual for Sleep; STrAtegies for RelaTives) for people with dementia and sleep disturbances: a single-blind feasibility and acceptability randomized controlled trial***

Characteristics of Research Subjects

| First author | Livingston |
| --- | --- |
| Year of publication | 2019 |
| Publication type | Journal |
| Number of patients (Intervention/Control) | 62 (42/20) |
| Age (Mean ± SD) | 80.4 ± 9/79.6 ± 7 |
| Gender (Male/Female) | I: 9/33  C: 10/10 |
| Country | UK |
| Measures for the intervention group | Morning bright light + sleep education |
| Measures for the control group | Treatment as usual (no light therapy) |
| Setting | Community |
| Light therapy | Intervention: Morning bright light + sleep education  Control: Treatment as usual (no light therapy) |
| MMSE score | Very mild, mild, moderate, severe AD with sleep disorders |
| Type of light therapy | Full spectrum white light |
| Light source / wave length | LED, NR |
| Light therapy device | Light box |
| Distance from light to patient | 25 cm in front of patient |
| Light intensity | 10000 lux |
| Intervention time | Same time every morning |
| Total time exposed to light therapy | 42 h |
| Follow-up time | NR |
| Outcomes | NPI, PSQI, ZBI, IS, IV, RA, SE |

| Continuous data  (pre-intervention) | Intervention | | | Control | | |
| --- | --- | --- | --- | --- | --- | --- |
|  | N | Means | SD | N | Means | SD |
| NPI | 41 | 42.02 | 23.17 | 20 | 46.90 | 23.48 |
| PSQI | 41 | 9.22 | 4.08 |  | 10.40 | 4.52 |
| ZBI | 42 | 37.69 | 18.39 |  | 38.30 | 19.27 |
| Continuous data  (post-intervention) | Intervention | | | Control | | |
|  | N | Means | SD | N | Means | SD |
| NPI | 36 | 38.69 | 23.57 | 18 | 44.72 | 23.22 |
| PSQI | 38 | 9.37 | 4.16 | 18 | 9.5 | 4.49 |
| ZBI | 38 | 36.5 | 17.07 | 19 | 42.16 | 16.65 |
| IS | 42 | 0.38 | 0.16 | 20 | 0.43 | 0.17 |
| IV | 42 | 1.11 | 0.4 | 20 | 1.03 | 0.32 |
| RA | 42 | 0.67 | 0.25 | 20 | 0.75 | 0.25 |
| SE | 42 | 75.5 | 12.1 | 20 | 79.2 | 11.5 |

***Study Title: Effect of home-based light treatment on persons with dementia and their caregivers***

Characteristics of Research Subjects

| First author | Sloane |
| --- | --- |
| Year of publication | 2015 |
| Publication type | Journal |
| Number of patients (Intervention/Control) | 17 (15/16) |
| Age (Mean ± SD) | 11 out of 17 participants >80 |
| Gender (Male/Female) | 6/11 |
| Country | USA |
| Measures for the intervention group | Whole day blue white light |
| Measures for the control group | Red−yellow light |
| Setting | Community |
| Light therapy | Intervention: Whole day blue white light  Control: Red−yellow light |
| MMSE score | 12.7 ± 9.1 |
| Type of light therapy | Blue enriched white light |
| Light source / wave length | LED, 470 nm |
| Light therapy device | Table, floor lamps, and light box |
| Distance from light to patient | NR |
| Light intensity | 300−400 lux |
| Intervention time | Awakening time to 18:00 |
| Total time exposed to light therapy | 504 h |
| Follow-up time | NR |
| Outcomes | IS, IV, SE, ZBI, CSDD |

| Continuous data  (post-intervention) | Intervention | | | Control | | |
| --- | --- | --- | --- | --- | --- | --- |
|  | N | Means | SD | N | Means | SD |
| SE | 15 | 68.4 | 21.6 | 16 | 68.9 | 20.3 |
| IS | 15 | 0.41 | 0.17 | 16 | 0.41 | 0.16 |
| IV | 15 | 1.21 | 0.30 | 16 | 1.20 | 0.27 |
